# Accelerated aging-related transcriptome alterations in neurovascular unit cells in the brain of Alzheimer’s disease

**DOI:** 10.3389/fnagi.2022.949074

**Published:** 2022-08-18

**Authors:** Yan Zhao, Yong-Zhi Xie, You-Shuo Liu

**Affiliations:** ^1^Department of Geriatrics, The Second Xiangya Hospital, Central South University, Changsha, China; ^2^Institute of Aging and Age-Related Disease Research, Central South University, Changsha, China; ^3^Department of Radiology, The Third Xiangya Hospital, Central South University, Changsha, China

**Keywords:** Alzheimer’s disease, aging, single-nucleus RNA sequencing, neurovascular unit, neurodegenerative disease

## Abstract

Alzheimer’s disease (AD) is the most common cause of dementia with no effective therapies. Aging is a dominant risk factor for AD. The neurovascular unit (NVU) plays an important role in maintaining homeostasis of the brain microenvironment. The accelerated aging of NVU cells may directly impair NVU function and contribute to AD pathogenesis. However, the expression patterns of aging-related genes (AGs) in NVU cells of AD remain unclear. In this study, we performed single-nucleus transcriptome analysis of 61,768 nuclei from prefrontal cortical samples of patients with AD and normal control (NC) subjects. Eight main cell types were identified, including astrocytes, microglia, excitatory neurons, inhibitory neurons, oligodendrocytes, oligodendrocyte precursor cells, pericytes, and endothelial cells. Transcriptomic analysis identified the expression patterns of AGs in NVU cells of AD. Gene set enrichment analysis confirmed the key aging-associated cellular pathways enriched in microglia and oligodendrocytes. These aging-related transcriptomic changes in NVU were cross-validated using bulk transcriptome data. The least absolute shrinkage and selection operator regression method was used to select the crucial AGs most associated with AD: IGF1R, MXI1, RB1, PPARA, NFE2L2, STAT5B, FOS, PRKCD, YWHAZ, HTT, MAPK9, HSPA9, SDHC, PRKDC, and PDPK1. This 15-gene model performed well in discriminating AD from NC samples. Among them, IGF1R, MXI1, PPARA, YWHAZ, and MAPK9 strongly correlated with pathologic progression in AD, were identified as critical regulators of AD. Although most AGs showed similar trends of expression changes in different types of NVU cells in AD, certain AGs were expressed in a cell-specific manner. Our comprehensive analysis of brain NVU from patients with AD reveals previously unknown molecular changes associated with aging that may underlie the functional dysregulation of NVU, providing important insights for exploring potential cell-specific therapeutic targets to restore brain homeostasis in AD.

## Introduction

Alzheimer’s disease (AD) is the most common form of dementia in the elderly population. Its pathological hallmarks include amyloid-beta (Aβ) plaques, neurofibrillary tangles, neuroinflammation, and loss of neurons and synapses (Fu et al., 2019). Despite a persistent search for the etiology and pathogenesis of AD, no effective treatment has been found, which may be partly due to our incomplete understanding of the molecular mechanism of cell type-specific responses underlying AD pathogenesis. A deeper investigation into cellular heterogeneity and cell type-specific responses might provide precise molecular and cellular targets for AD treatment.

The brain neurovascular unit (NVU), which includes neurons, glial cells, vascular cells, and pericytes, is an important functional unit involved in the maintenance of brain homeostasis through regulation of neurovascular coupling (Iadecola, 2017). The structure and function of NVU are profoundly impaired in AD (Iadecola, 2004; Zlokovic, 2011). In contrast to the neurocentric view, the neurovascular concept proposes that disintegration of NVU function may be a major cause of dementia in the elderly population, and also a culprit in AD (Zlokovic, 2008b). Neurovascular uncoupling can lead to blood brain barrier (BBB) leakage, vascular inflammation, cerebral hypoperfusion, and Aβ accumulation around cerebral vessels (Zlokovic, 2008a; Wilhelm et al., 2017; Armstead and Vavilala, 2019). The aging process, a progressive decline of internal physiological function over time, is a dominant risk factor for many chronic diseases, including AD. The effects of aging on NVU function are evident. First, significant evidence has been reported that aging can induce BBB dysfunction by altering molecular and signal transduction within the NVU (Montagne et al., 2015; Nelson et al., 2016). Age-dependent endothelial impairment is a major contributor to BBB dysfunction. Aging impairs the regenerative capacity of endothelial cells (ECs) (Lähteenvuo and Rosenzweig, 2012) and the endothelium-dependent regulation of vascular tone (Hu et al., 2017). Aging also enhances the sensitivity of ECs toward apoptotic stimuli, further contributing to the decrease in cell number (Shantsila et al., 2007). Moreover, brain ECs have robust intrinsic antioxidative defenses, which can be damaged by aging (Deli et al., 2005). Second, senescent microglia tend to transform from an anti-inflammatory M2 phenotype into an proinflammatory M1 phenotype (Lourbopoulos et al., 2015). The oxidation and nitration injury from the microglial proinflammatory response leads to a vicious cycle of continued microglial activation and oxidative neural injury (Zhan et al., 2005). Third, the function of other NVU cells, including neurons, astrocytes, and oligodendrocytes are also compromised with aging (Hu et al., 2017). Senescent cells acquire the ability to secrete a variety of cytokines, such as pro-inflammatory factors, metalloproteinases, and other proteins, known as senescence-associated secretory phenotype (SAPS) (Tchkonia et al., 2013). Senescent neural cells contribute to neuroinflammation by secreting large amounts of proinflammatory mediators during aging (Campisi, 2013), whereas, inflammation is a central factor affecting NVU function during aging (Wilhelm et al., 2017). The structure and function of NVU are influenced by aging; However, the expression characteristics of aging-related genes (AGs) and possible regulatory mechanisms of NVU dysfunction in AD remain unclear.

The Human Aging Genomic Resources (HAGR) is a database specifically addressing aging-related changes that has revealed a set of AGs *via* systemic analysis of the biology and genetics of the human aging process (de Magalhães et al., 2005). To comprehensively investigate the aging-related transcriptomic changes and dysregulated molecular pathways in each NVU cell type in AD, we performed single-nucleus RNA-sequencing (snRNA-seq) analysis of the AD and normal control (NC) brain samples. These aging-related changes in NVU were further validated in AD brain tissue using microarray data. To identify the AGs that were most associated with AD, we fitted a least absolute shrinkage and selection operator (LASSO) regression model for feature selection. A 15-gene-based model was constructed and tested in a test set and a validation set. We next analyzed the expression levels of these selected AGs in patients with different Braak stages and plaque scores to identify critical regulators of AD. Finally, we analyzed the cell type-specific expression of these key genes. The advent of snRNA-seq techniques contributes to more comprehensive knowledge about the alterations in NVU. In-depth investigation of the cell type-specific molecular alterations in the AD brain might provide precise cellular targets for AD therapeutic development.

## Materials and methods

### Single-nucleus RNA sequencing data acquisition and processing

The Gene Expression Omnibus (GEO) is a public genomics dataset repository collecting chips, microarrays and high-throughput sequencing data (Edgar et al., 2002). The snRNA-seq data of a total of 61,768 cells of 11 AD samples and seven normal control samples, accession number GSE174367 (Morabito et al., 2021), were downloaded from the GEO database. The snRNA-seq data were analyzed using the “Seurat” R package (Stuart et al., 2019). To exclude potential low quality cells, nuclei with ≤200 genes, ≥6,000 genes, or ≥15% mitochondrial genes were filtered out, as described in previous snRNA-seq studies (Jäkel et al., 2019; Morabito et al., 2021). A total of 60,766 nuclei were retained after filtration. The filtered matrix was normalized using the “NormalizeData” method. Then, we identified the top 2,000 highly variable genes (HVGs) for each sample using the “FindVariableFeatures” function with the “vst” method. Genes with the highest variance were used to perform principal components analysis (PCA), and the number of principal components used in downstream analyses was chosen using the “Elbowplot.” Twenty principal components (PCs) were used for the uniform manifold approximation and projection (UMAP) analysis. Cells were clustered into 25 clusters, with resolution set at 0.5, using the functions “FindNeighbors” and “FindClusters.” Differentially expressed genes (DEGs) for each cluster were identified using the function “FindAllMarkers” with the parameters test.use = wilcox and logfc.threshold = 0.25. The identification of cell type was based on the DEGs in each cluster and classical cell markers as previously reported (Lau et al., 2020). To define the cell type-specific transcriptomic changes in AD, we compared the transcriptome profiles of each cell type between NC and AD samples using the “FindMarkers” function. Significance was set at a *p*-value < 0.05 and a | log2 Fold Change(FC)| > 0.1. We visualized the data using Seurat’s Doheatmap or DotPlot function where appropriate.

### Bulk transcriptome data acquisition and processing

Four microarray datasets (GSE48350, GSE122063, GSE106241, and GSE29378) of AD were obtained from the GEO database of National Center for Biotechnology Information (NCBI). Data for 80 AD patients and 173 control samples from GSE48350, 56 AD patients and 44 healthy controls from GSE122063, 31 AD patients and 32 controls from GSE29378, and 60 AD cases with multiple clinical information about AD severity from GSE106241 were analyzed in this study. All datasets enrolled in the study are listed in Table 1. For microarray data processing, DEGs between AD and NC brain tissues were identified by the “limma” R package (Ritchie et al., 2015), with screening criteria of adjusted *p*-value < 0.05 and | log2 FC| > 0.1. DEG volcano plot was depicted using the “EnhancedVolcano” R packages.

**TABLE 1:** The enrolled datasets in the study.

Datasets	Type	Platform	Sample size (Control/AD)	Cells (Control/AD)	Sample source	References
GSE174367	snRNA sequencing	GPL24676 Illumina NovaSeq 6000 (Homo sapiens)	7/11	22901/38867	Prefrontal cortex	Morabito et al., 2021
GSE48350	Microarray	GPL570 Affymetrix Human Genome U133 Plus 2.0 Array	173/80	-	Hippocampus, entorhinal cortex, superior frontal cortex, post-central gyrus	Berchtold et al., 2008
GSE122063	Microarray	GPL1669 Agilent-039494 SurePrint G3 Human GE v2 8x60K Microarray 039381 (Feature Number version)	44/56	-	Frontal cortex, temporal cortex	McKay et al., 2019
GSE29378	Microarray	GPL6947 Illumina HumanHT-12 V3.0 expression beadchip	32/31	-	Hippocampus	Miller et al., 2013
GSE106241	Microarray	GPL24170 Agilent-044312 Human 8x60K Custom Exon array (Probe Name version)	0/60	-	Temporal cortex	Marttinen et al., 2019

The first dataset was used in the snRNA-seq analysis of distinct NVU cell types to identify cell type-specific transcription. The second dataset was used for LASSO regression analysis to screen the most crucial genes for AD diagnosis. The third dataset was used for independent validation analysis. The last two datasets were used to evaluated the correlation between key AGs and AD severity. AD, Alzheimer’s disease; GPL, gene expression omnibus platform.

### Pathway and functional enrichment analysis

We performed gene set enrichment analysis (GSEA) to explore the potential pathways involved in AD pathological processes, and the significant gene sets were defined as those with a normalized enrichment score (NES) > 1 and *p* < 0.05 (Subramanian et al., 2005). Gene Ontology (GO) and Kyoto Encyclopedia of Genes and Genomes (KEGG) enrichment analyses of DEGs were performed to analyze their overall functions, with *q*-value < 0.05 considered significant for screening of significant GO and KEGG pathways (Kanehisa et al., 2016; Consortium, 2019). The functional enrichment analyses were performed using R package “ReactomeRA” and “clusterProfiler” (Yu et al., 2012; Yu and He, 2016).

### Cross-study validation of aging-related changes with bulk transcriptome data

Human aging genomic resources provide databases associated with the study of aging. A total of 307 human AGs were acquired from the HAGR dataset^1^ (Tacutu et al., 2018; Supplementary Table 1). We overlapped differentially expressed aging-related genes (DEAGs) in NVU cells (from the snRNA-seq dataset GSE174367) with those from brain tissues between AD and NC (from the microarray dataset GSE48350). The Venn diagram was used to depict the overlap between DEG transcripts.

### Construction and validation of the least absolute shrinkage and selection operator cox regression model

We performed LASSO regression (Simon et al., 2011), a method designed for variable selection and shrinkage, to identify the AGs most associated with AD. The samples from GSE48350 were randomly assigned to the training set (70%) or test set (30%). The overlapped DEAGs were extracted and fit into the LASSO regression model by the “glmnet” package. To assess the ability of key AGs to differentiate between AD and NC, receiver operating characteristic (ROC) analysis was completed using the “ROCR” package in the test and external validation sets (GSE122063). Models with an area under the curve (AUC) value greater than 0.7 were considered to be good classifier models.

### Correlation of aging-related genes expression levels with Alzheimer’s disease severity

We next assessed the expression levels of the important AGs in NVU cells. We validated the AG expressions in different Braak stages using the brain samples in the dataset GSE106241. We also compared the expression levels of AGs in patients with different plaque scores in the dataset GSE29378. Differences between the two groups were compared using the two-tailed unpaired *t*-test. The boxplots were created using the R package “ggplot2.”

## Results

### Single-nucleus transcriptome profiling of neurovascular unit cells in Alzheimer’s disease

**FIGURE 1:**
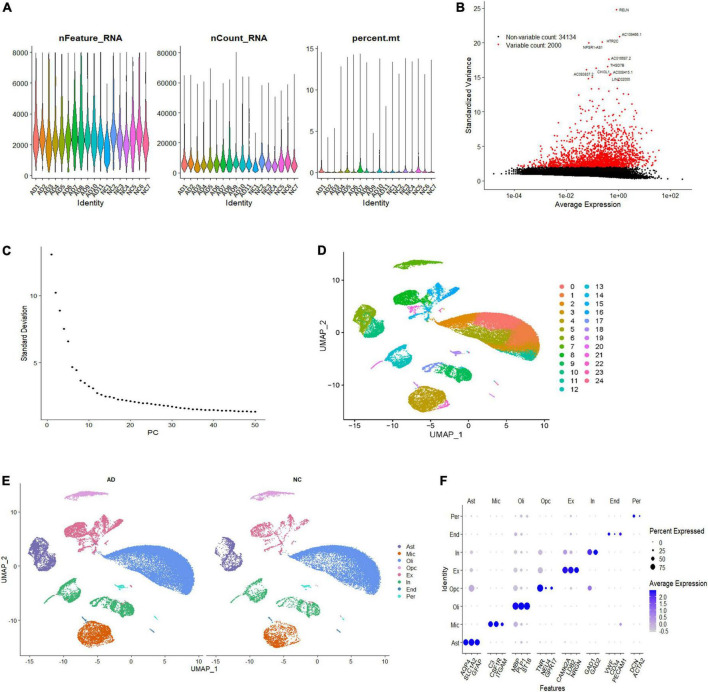
Single-nucleus transcriptome analysis of the human AD prefrontal cortex. **(A)** The gene features, counts, and mitochondrial gene percentage of each sample. **(B)** HVGs were identified and colored in red, and the top ten HVGs were labeled. **(C)** PC selection using Elbowplot function. **(D)** UMAP plot analysis showing 25 major cell clusters based on gene expression profile. **(E)** UMAP projection of overall cell types of AD and NC samples, respectively. Different cell types were colored with unique colors. **(F)** Dot plot of marker gene expression of each cell type. The dot size represents the percentage of cells expressing selected genes, and the dot color represents average expression. AD, Alzheimer’s disease; Ast, astrocyte; End, endothelial cell; Ex, excitatory neuron; HVGs, highly variable genes; In, inhibitory neuron; Mic, microglia; NC, normal control; Oli, oligodendrocyte; Opc, oligodendrocyte progenitor cell; PC, primary component; Per, pericyte; UMAP, uniform manifold approximation and projection.

To investigate the cell-specific transcriptional features in the brain of patients with AD, we analyzed the single-nucleus transcriptome data of 18 prefrontal cortex tissue samples from patients with AD (*n* = 11) and NC subjects (*n* = 7). A total of 60,766 nuclei were retained after quality control filtration, comprising 38,179 nuclei from AD patients and 22,587 from NC. The expression characteristics of each sample are shown in Figure 1A. After 2,000 HVGs were identified (Figure 1B), PCA was performed, and the top 20 PCs were used for clustering (Figure 1C). The UMAP plot was used to visualize the data in a two-dimensional subspace, identifying 25 cell clusters with highly consistent expression patterns across individuals (C0-C24) (Figure 1D). We categorized these cell clusters into eight major cell types according to their respective transcriptional signatures and cell-type markers reported in previous literature: astrocytes, microglia, oligodendrocytes, oligodendrocyte precursor cells (OPCs), excitatory neurons, inhibitory neurons, ECs, and pericytes (Figure 1E). The expression of cell-type markers is exhibited in Figure 1F. The identified marker genes of astrocytes (AQP4, SLC1A2, GFAP), microglia (C3, CSF1R, ITGAM), oligodendrocytes (PLP1, ST18, MBP), OPCs (TNR, NEU4, GPR17), excitatory neurons (CAMK2A, LDB2, NRGN), inhibitory neurons (GAD1, GAD2), ECs (VWF, CD34, PECAM1), and pericytes (DCN, ACTA2) were cell type-specific and in accordance with classic markers for each cell population (Lau et al., 2020; Srinivasan et al., 2020). The cell-type proportion is comparable to those reported in previous snRNA-seq studies of the brain (Morabito et al., 2021).

### Single-nucleus RNA-sequencing reveals the heterogeneity of the aging-associated transcriptome and dysregulated molecular pathways in Alzheimer’s disease

**FIGURE 2:**
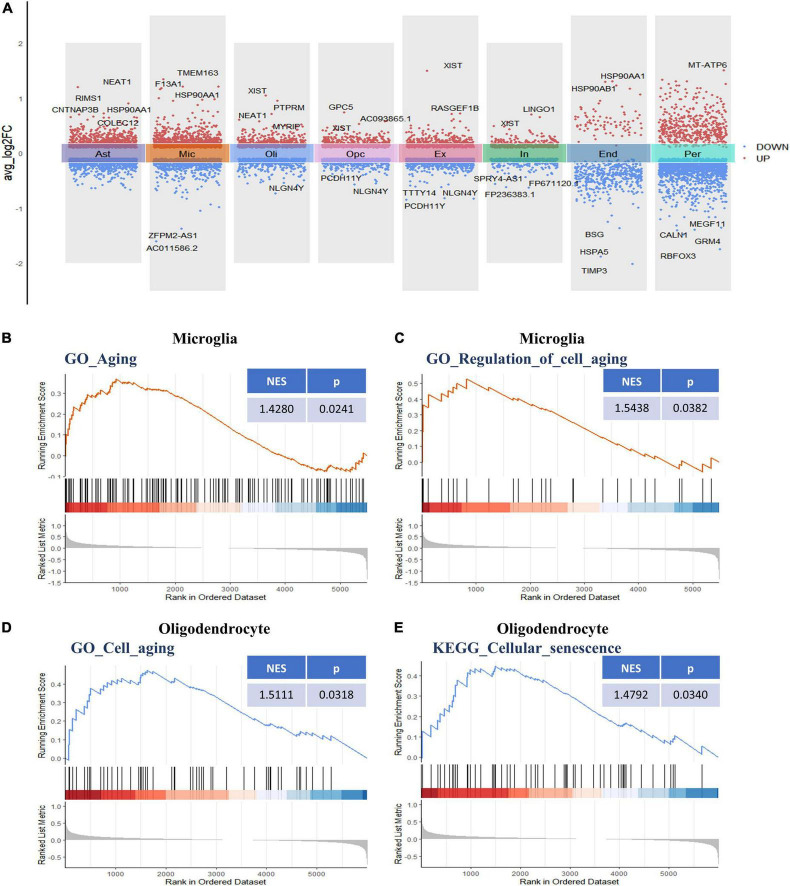
Differential gene expression and gene set enrichment analysis. **(A)** Differential gene expression analysis showing up- and downregulated genes across all the eight cell types (*p* < 0.05, |log2 FC| > 0.1). Red dots represent upregulated genes, and blue dots represent downregulated genes. The colored bars represent different cell types. **(B)** GSEA enrichment plot of the “GO-BP: Aging (GO: 0007568)” gene set in microglia. **(C)** GSEA enrichment plot of the “GO-BP: Regulation of cell aging (GO: 0090342)” gene set in microglia. **(D)** GSEA enrichment plot of the “GO-BP: Cell aging (GO: 0007569)” gene set in oligodendrocytes. **(E)** GSEA enrichment plot of the “KEGG: Cellular senescence (hsa04218)” gene set in oligodendrocytes. BP, biological process; GO, gene ontology; GSEA, gene set enrichment analysis; KEGG, Kyoto encyclopedia of genes and genomes.

Following initial cell-type characterization, we compared the individual cell-type transcriptional profile between AD and NC samples separately. Between the AD and NC brain samples, we found the following number of DEGs per cell type: astrocytes (*n* = 1,908), microglia (*n* = 1,753), oligodendrocytes (*n* = 1,128), OPCs (*n* = 1,186), ECs (*n* = 493), excitatory neuron (*n* = 1,152), inhibitory neurons (*n* = 683), and pericytes (*n* = 2,110) (Figure 2A and Supplementary Table 2). The changes in molecular phenotypes might help to explain the functional alterations in individual cell types; thus, we performed GSEA analysis to investigate the potential biological roles of the DEGs in each NVU cell type. In GSEA analysis, we observed DEGs in microglia and oligodendrocytes partly enriched in cellular senescence-related pathways. Specifically, the GO gene sets analyzed by GSEA revealed that the biological processes (BP) associated with DEGs includes aging, regulation of cell aging, and cell aging processes (Figures 2B–D). For KEGG pathway analysis, dysregulated genes were mainly enriched in the cellular senescence pathway (Figure 2E). The complete results of GSEA analysis can be found in Supplementary Table 3.

**FIGURE 3:**
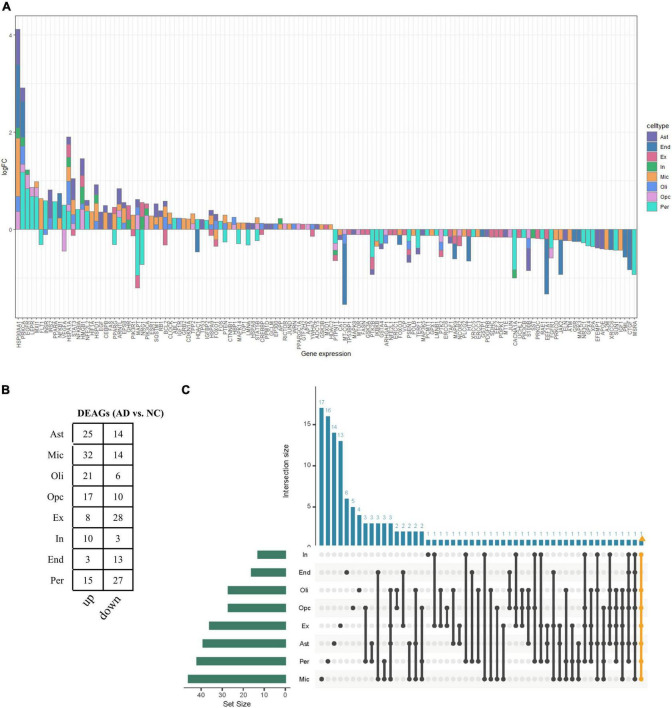
Aging-associated transcriptomic changes in NVU cells. **(A)** Consensus DEAGs upregulated (positive log2 FC) or suppressed (negative log2 FC) in NVU cells (*p* < 0.05, | log2 FC| > 0.1). **(B)** Numbers of DEAGs between AD and NC samples within each cell type. Down: downregulated; up: upregulated. **(C)** UpSet plot depicting the number of overlap DEAGs across all eight cell types. Also shown are the numbers of DEAGs specific to each cell type. AD, Alzheimer’s disease; Ast, astrocyte; DEAGs, differentially expressed aging-related genes; End, endothelial cell; Ex, excitatory neuron; FC, fold change; In, inhibitory neuron; Mic, microglia; NC, normal control; Oli, oligodendrocyte; Opc, oligodendrocyte progenitor cell; Per, pericyte.

The functional changes of NVU in AD may be in part attributable to the expression alterations in AGs. A total of 307 human AGs were obtained from HAGR (Supplementary Table 1). To acquire the DEAGs in each NVU cell type, we overlapped the DEGs in different NVU cell types with the AGs, respectively. The number of filtered DEAGs per cell type were as follows: astrocytes (*n* = 39), microglia (*n* = 46), oligodendrocytes (*n* = 27), OPCs (*n* = 27), excitatory neurons (*n* = 36), inhibitory neurons (*n* = 13), ECs (*n* = 16), and pericytes (*n* = 42) (Figures 3A,B and Supplementary Table 4). Of interest was that most DEAGs in the NVU cells were found to be simultaneously up- or downregulated. Notably, the DEAGs among different cell types varied significantly. Detailed information about the intersections of DEAGs among different NVU cell types was visualized using an UpSet plot (Figure 3C). In particular, we found that none of the DEAGs were existed in all NVU cell types, and only one common DEAG was present in seven of the eight cell types (except for ECs), indicating that different cell types are heterogeneously affected by aging. Aging-related transcriptomic changes in NVU are cell type-specific, thus it is necessary to disclose the role of AGs in AD pathogenesis at the single cell level. While the snRNA-seq analysis would enable us to identify the target cell types, we sought to additionally identify the potential target genes for AD.

### Identification of key aging-related genes in Alzheimer’s disease using the least absolute shrinkage and selection operator cox regression model

**FIGURE 4:**
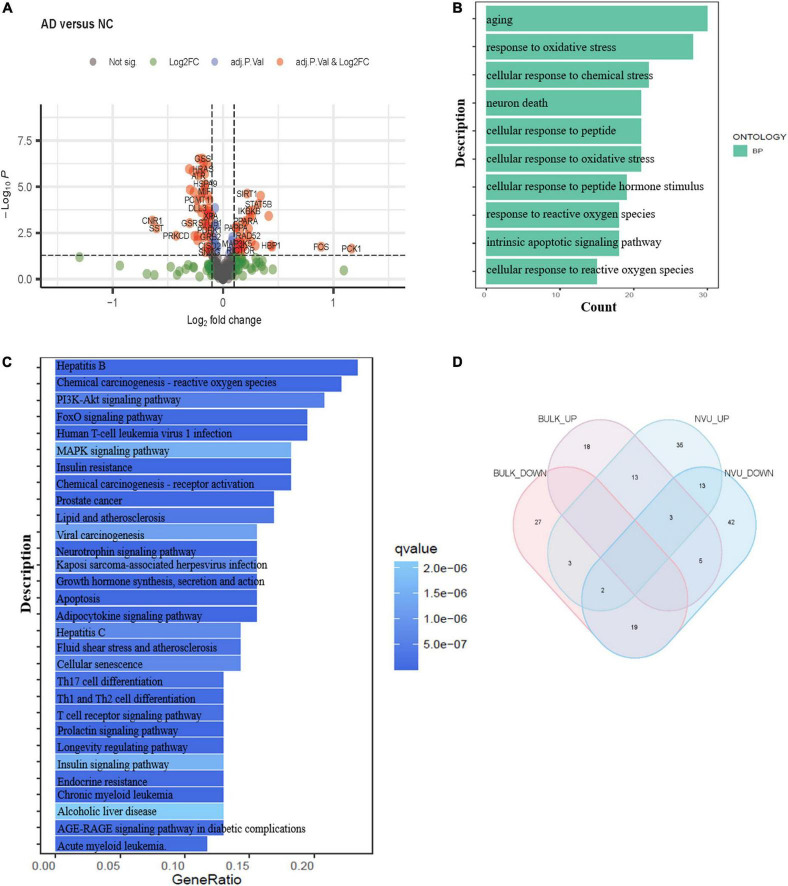
Validation of the aging-related transcriptomic changes in the AD brain based on data from a microarray study. **(A)** The volcano plot of DEAGs in GSE48350 (adjusted *p*-value < 0.05 and | log2 FC| > 0.1). **(B)** The GO analysis of DEAGs in GSE48350. **(C)** The KEGG pathway analysis of DEAGs in GSE48350. **(D)** Veen plot showed the common downregulated DEAGs and common upregulated DEAGs between NVU cells (from snRNA-seq dataset: GSE174367) and brain tissues (from bulk transcriptome dataset: GSE48350). A total of 37 common DEAGs were found, including 16 upregulated genes and 21 downregulated genes. AD, Alzheimer’s disease; DEAGs, differentially expressed aging-related genes; GO, gene ontology; KEGG, Kyoto encyclopedia of genes and genomes; NVU, neurovascular unit.

Bulk transcriptome analysis is helpful for collecting key information about cell populations and screening features genes. To validate the aging-associated transcriptomic changes in NVU of AD detailed above, we compared our results with the bulk transcriptome dataset GSE48350. A total of 90 DEAGs, including 39 upregulated genes and 51 downregulated genes, were obtained based on an adjusted *p*-value < 0.05 and | log2 Fold Change| > 0.1 (Supplementary Table 5). The DEAGs were visualized using the volcano plot (Figure 4A). GO and KEGG were performed to depict the biological processes and molecular pathways of the DEAGs. These genes were significantly enriched in cellular senescence, longevity regulating pathway, and apoptosis pathway (Figures 4B,C and Supplementary Table 6). Among the 153 DEAGs identified in our snRNA-seq analysis, 37 DEAGs in the brain cortex microarray data exhibited concordant changes in expression levels (Figure 4D and Supplementary Table 7), of which 16 genes were upregulated, and 21 genes were downregulated.

**FIGURE 5:**
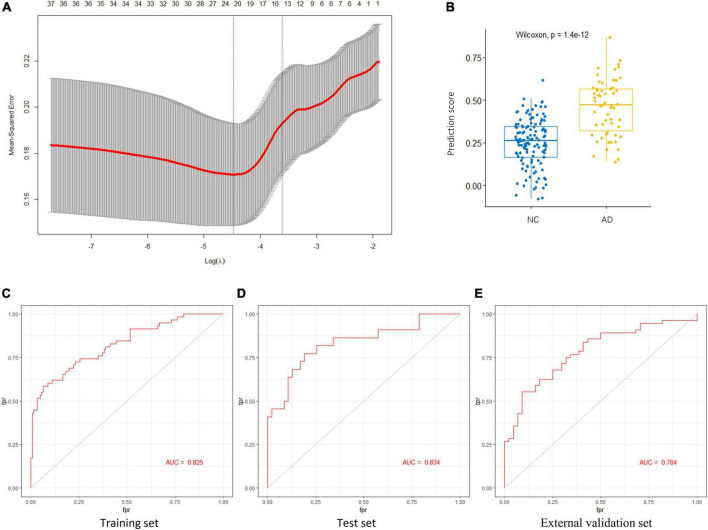
Key AGs selection through the LASSO model. **(A)** The selection of the best lambda in the LASSO model. **(B)** Boxplot used to assess the performance of the prediction model. **(C)** The AUC was 0.825 in training set. **(D)** The AUC was 0.834 in test set. **(E)** The AUC was 0.784 in validation set GSE122063. AG, aging-related gene; AUC, area under the curve; LASSO, least absolute shrinkage and selection operator.

To identify the AGs most associated with AD, the 37 DEAGs were entered into a LASSO logistic regression model. We separated all samples in GSE48350 (80 AD and 173 NC samples) into training and test sets. Fifteen potential predictors in the training set were identified to construct a model to calculate the risk score in the LASSO logistic regression (Figure 5A). The coefficient values and information of the 15 genes are displayed in Table 2. We calculated a risk score for each patient based on the expression of the 15 genes using the LASSO regression model:

**TABLE 2:** The 15 genes associated with the risk model in Alzheimer’s disease (AD).

ENSG ID	Symbol	Location	Expression status	Coefficient
ENSG00000140443	IGF1R	Chromosome 15	Upregulated	0.0454
ENSG00000119950	MXI1	Chromosome 10	Upregulated	–0.1831
ENSG00000139687	RB1	Chromosome 13	Upregulated	0.2731
ENSG00000186951	PPARA	Chromosome 22	Upregulated	0.1343
ENSG00000116044	NFE2L2	Chromosome 2	Upregulated	0.0939
ENSG00000173757	STAT5B	Chromosome 17	Upregulated	0.0395
ENSG00000170345	FOS	Chromosome 14	Upregulated	0.0085
ENSG00000163932	PRKCD	Chromosome 3	Downregulated	–0.0081
ENSG00000164924	YWHAZ	Chromosome 8	Downregulated	–0.0109
ENSG00000197386	HTT	Chromosome 4	Downregulated	–0.0957
ENSG00000050748	MAPK9	Chromosome 5	Downregulated	0.0422
ENSG00000113013	HSPA9	Chromosome 5	Downregulated	–0.1732
ENSG00000143252	SDHC	Chromosome 1	Downregulated	–0.0575
ENSG00000253729	PRKDC	Chromosome 8	Downregulated	–0.3421
ENSG00000140992	PDPK1	Chromosome 16	Downregulated	–0.0006


r⁢i⁢s⁢k⁢s⁢c⁢o⁢r⁢e=(0.0454⁢e⁢x⁢p⁢r⁢e⁢s⁢s⁢i⁢o⁢n⁢v⁢a⁢l⁢u⁢e⁢o⁢f⁢I⁢G⁢F⁢1⁢R)+(0.2731×e⁢x⁢p⁢r⁢e⁢s⁢s⁢i⁢o⁢n⁢v⁢a⁢l⁢u⁢e⁢o⁢f⁢R⁢B⁢1)+(0.1343×e⁢x⁢p⁢r⁢e⁢s⁢s⁢i⁢o⁢n⁢v⁢a⁢l⁢u⁢e⁢o⁢f⁢P⁢P⁢A⁢R⁢A)+(0.0939×e⁢x⁢p⁢r⁢e⁢s⁢s⁢i⁢o⁢n⁢v⁢a⁢l⁢u⁢e⁢o⁢f⁢N⁢F⁢E⁢2⁢L⁢2)+(0.0395×e⁢x⁢p⁢r⁢e⁢s⁢s⁢i⁢o⁢n⁢v⁢a⁢l⁢u⁢e⁢o⁢f⁢S⁢T⁢A⁢T⁢5⁢B)+(0.0085×e⁢x⁢p⁢r⁢e⁢s⁢s⁢i⁢o⁢n⁢v⁢a⁢l⁢u⁢e⁢o⁢f⁢F⁢O⁢S)+(0.0422×e⁢x⁢p⁢r⁢e⁢s⁢s⁢i⁢o⁢n⁢v⁢a⁢l⁢u⁢e⁢o⁢f⁢M⁢A⁢P⁢K⁢9)-(0.1831×e⁢x⁢p⁢r⁢e⁢s⁢s⁢i⁢o⁢n⁢v⁢a⁢l⁢u⁢e⁢o⁢f⁢M⁢X⁢I⁢1)-(0.0081×e⁢x⁢p⁢r⁢e⁢s⁢s⁢i⁢o⁢n⁢v⁢a⁢l⁢u⁢e⁢o⁢f⁢P⁢R⁢K⁢C⁢D)-(0.0109×e⁢x⁢p⁢r⁢e⁢s⁢s⁢i⁢o⁢n⁢v⁢a⁢l⁢u⁢e⁢o⁢f⁢Y⁢W⁢H⁢A⁢Z)-(0.0957×e⁢x⁢p⁢r⁢e⁢s⁢s⁢i⁢o⁢n⁢v⁢a⁢l⁢u⁢e⁢o⁢f⁢H⁢T⁢T)-(0.1732×e⁢x⁢p⁢r⁢e⁢s⁢s⁢i⁢o⁢n⁢v⁢a⁢l⁢u⁢e⁢o⁢f⁢H⁢S⁢P⁢A⁢9)-(0.0575×e⁢x⁢p⁢r⁢e⁢s⁢s⁢i⁢o⁢n⁢v⁢a⁢l⁢u⁢e⁢o⁢f⁢S⁢D⁢H⁢C)-(0.3421×e⁢x⁢p⁢r⁢e⁢s⁢s⁢i⁢o⁢n⁢v⁢a⁢l⁢u⁢e⁢o⁢f⁢P⁢R⁢K⁢D⁢C)-(0.0006×e⁢x⁢p⁢r⁢e⁢s⁢s⁢i⁢o⁢n⁢v⁢a⁢l⁢u⁢e⁢o⁢f⁢P⁢D⁢P⁢K⁢1)

The LASSO regression model based on the 15 AGs could efficiently distinguish patients with AD from NC subjects (*p* < 1.4e-12, Wilcoxon test) (Figure 5B). The AUC for the ROC curve of the model was 0.825 in the training set (Figure 5C) and 0.834 in the test set (Figure 5D). Subsequently, we evaluated the ability of the LASSO regression model to distinguish between AD and NC samples in the external validation set, and the AUC of the model was 0.784 (Figure 5E). These results demonstrate that our 15-gene-based model displays a relatively good capacity to classify AD and NC samples.

### Validation of aging-associated transcriptomic changes in multiple cortical regions

Subsequently, we validated the expression of the 15 key AGs across multiple brain regions (in GSE48350). Among the 15 AGs identified before, 11 genes were differentially expressed in the superior frontal cortex (AD: *n* = 21, NC: *n* = 48), 9 in the hippocampus (AD: *n* = 19, NC: *n* = 43), 10 in the entorhinal cortex (AD: *n* = 15, NC: *n* = 39), and eight in the post-central gyrus (AD: *n* = 25, NC: *n* = 43) (Supplementary Figure 1). These replicable DEAGs in various brain regions exhibited concordant changes in expression levels. These results further validated the aging-associated transcriptomic changes identified in snRNA-seq analysis.

### The correlation between dysregulated aging-related genes and Alzheimer’s disease severity

**FIGURE 6:**
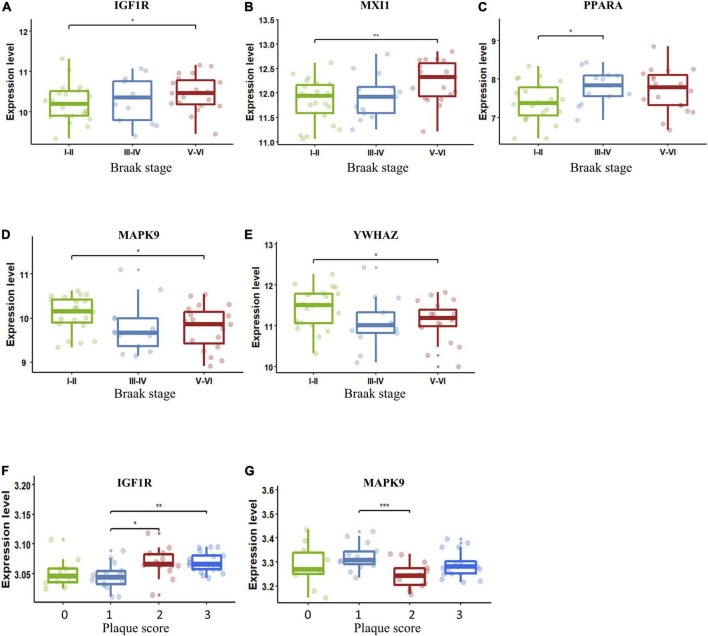
Key AG expression levels in AD patients with different Braak stages or plaque scores. **(A)** The expression of IGF1R in different Braak stages of AD. **(B)** The expression of MXI1 in different Braak stages of AD. **(C)** The expression of PPARA in different Braak stages of AD. **(D)** The expression of MAPK9 in different Braak stages of AD. **(E)** The expression of YWHAZ in different Braak stages of AD. **(F)** The expression of IGF1R in different plaque scores of AD. **(G)** The expression of MAPK9 in different plaque scores of AD. The upper, lower, and middle horizontal lines of the box represent the upper, lower, and median quartiles, respectively. The *t*-test was used to estimate whether differences between the two groups were significant. Asterisks denotes significant difference. *p*-value: *p < 0.05; **p < 0.01; ***p < 0.001. AD, Alzheimer’s disease; AG, aging-related gene.

The importance of the key AGs in AD has been confirmed. We next sought to determine if the change in these AGs correlated with indicators of AD severity. The presence of AD-type neurofibrillary lesions was quantified by Braak staging (Braak and Braak, 1991). We used samples in the dataset GSE106241 to examine whether the AG expressions varied in different Braak stages in AD. IGF1R and MXI1 were significantly upregulated in Braak V and VI compared with Braak I and II (Figures 6A,B). PPARA was significantly upregulated in Braak III and IV compared with Braak I and II (Figure 6C). MAPK9 and YWHAZ were identified to be downregulated in Braak V and VI compared with Braak I and II (Figures 6D,E). Amyloid plaque is another important pathological hallmark of AD, and higher amyloid plaque burden scores (0–3) indicate greater pathology. We analyzed the dataset GSE29378 to determine whether the expression levels of AGs varied in patients with different plaque scores. IGF1R was upregulated in patients with plaque score = 2/3 compared with those with plaque score = 1 (Figure 6F). MAPK9 was downregulated in patients with plaque score = 2 compared with those with plaque score = 1 (Figure 6G). Taken together, upregulated IGF1R, MXI1, and PPARA, and downregulated MAPK9 and YWHAZ in NVU cells may be involved in progression of AD pathology.

### Cell type-specific alterations of key aging-related genes in Alzheimer’s disease

**FIGURE 7:**
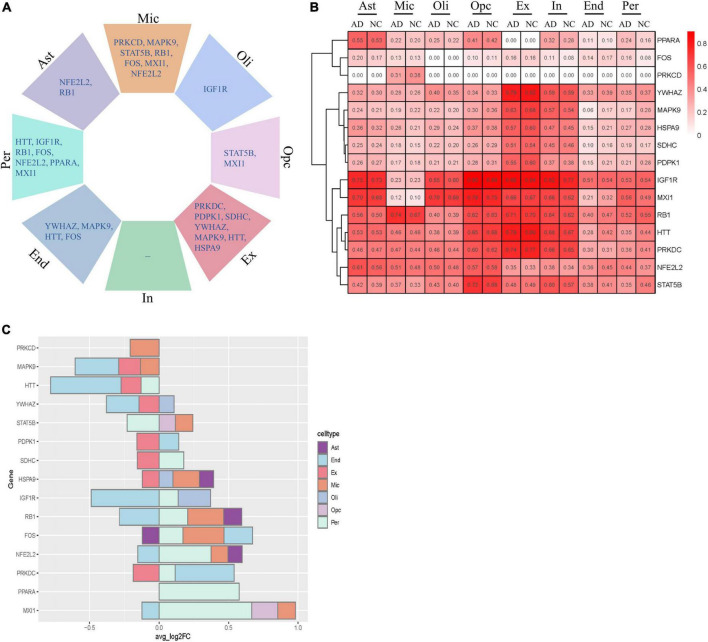
Cell type-specific alterations of key AGs in AD. **(A)** Key DEAGs in different NVU cell types. **(B)** The expression levels of key AGs in each NVU cell type. **(C)** Differentially expressed key AGs in NVU cells between AD and NC. AD, Alzheimer’s disease; AGs, aging-related genes; Ast, astrocyte; DEAGs, differentially expressed aging-related genes; End, endothelial cell; Ex, excitatory neuron; FC, fold change; In, inhibitory neuron; Mic, microglia; NC, normal control; NVU, neurovascular unit; Oli, oligodendrocyte; Opc, oligodendrocyte progenitor cell; Per, pericyte.

We next analyzed the variation in the expression of key AGs in each NVU cell type. These AGs were significantly altered in a specific cell type, including changes specific to astrocytes (e.g., NFE2L2 and RB1), microglia (e.g., PRKCD, MAPK9, STAT5B, RB1, FOS, MXI1, and NFE2L2), oligodendrocytes (e.g., IGF1R), OPCs (e.g., STAT5B and MXI1), excitatory neurons (e.g., PRKDC, PDPK1, SDHC, YWHAZ, MAPK9, HTT, and HSPA9), ECs (e.g., YWHAZ, MAPK9, HTT, and FOS), and pericyte (e.g., HTT, IGF1R, RB1, FOS, NFE2L2, PPARA, and MXI1) (Figure 7A). Figure 7B showed the relative expression profiles of each gene in each cell types. The key AGs show considerable heterogeneity among the cell type-specific expression patterns (Figure 7C). Bulk transcriptome data cannot detect DEGs with opposite directionality in different cell types. We previous found that upregulated IGF1R, MXI1, and PPARA, and downregulated MAPK9 and YWHAZ may be correlated with AD progression. With snRNA-seq analysis, we found that IGF1R was upregulated in oligodendrocytes and pericytes but downregulated in ECs. MXI1 was upregulated in microglia, pericytes and OPCs but downregulated in ECs. YWHAZ was downregulated in ECs, excitatory neurons but upregulated in oligodendrocytes. The single cell-level resolution is crucial as changes in gene expression, for example directionality, may be conditional on cell type.

## Discussion

Aging is one of the strongest risk factors for the development of AD. Aging may directly affect cells of NVU. However, scarce attention has been paid to the role of NVU aging in AD pathogenesis. Thus, defining the contribution of aging-related molecular pathways in NVU cells, and their role in the development of AD is of great importance. To address this question, we analyzed single-nucleus transcriptome data of prefrontal cortex from 11 AD patients and seven NC individuals. We examined the transcriptomes of each NVU cell type, including astrocytes, microglia, oligodendrocytes, OPCs, excitatory neurons, inhibitory neurons, ECs, and pericytes, to determine whether the eight cell types were impacted by aging. GSEA analysis confirmed the cellular senescence-associated pathways enriched in microglia and oligodendrocytes. The results consistent with previous findings that glia cells are more likely to be influenced by aging. Senescent microglia and astrocytes accumulate in MAPTP^301S^PS19 mouse model (a model of tau-dependent neurodegenerative disease), and clearance of these cells prevents neurofibrillary tangle deposition, gliosis, and degeneration of hippocampal and cortical neurons (Bussian et al., 2018). Oligodendrocytes are the largest group of non-neuronal cells in the brain, and the number of oligodendrocytes declines by age, which showed a significant 27% decrease over adult life from 20 to 90 years (Pelvig et al., 2008). Several Additional studies confirmed that oligodendrocytes in aging is associated with diminished remyelination capacity, increased atrophy of white matter, and a decline in motor learning (Ruckh et al., 2012; Habes et al., 2016).

To comprehensively investigate the transcriptional changes associated with aging in NVU, we next analyzed the expression patterns of AGs in each NVU cell type. Subsequently, validation of the aging-related transcriptomic changes in NVU was conducted in bulk transcriptomic data of AD. GO and KEGG pathway analysis in bulk transcriptomic data uncovered function of DEAGs, such as aging, apoptosis, cellular senescence, longevity regulating pathway. Although the relationships between aging and apoptosis are complex, studies have speculated that apoptosis contributes to aging (Zheng et al., 2005). Longevity is modulated by aging, and senescence inhibition leads to health and longevity (Tousian et al., 2020).

Using the LASSO method, we selected 15 crucial AGs based on the intersection of DEAGs from snRNA-seq data and those from bulk transcriptome data of AD, which performed well in discriminating AD from NC samples. Additionally, this 15-gene model was well-validated in the external dataset. Some of these genes have been reported previously to be involved in AD pathogenesis. It has been confirmed that aberrant IGF1R signaling existed in the post-mortem brains of AD patients, and long-term suppression of IGF1R signaling delayed AD progression. Genetic ablation of IGF1R in neurons of the aging mouse brain could protect against the neuroinflammation and memory impairment induced by Aβ oligomers (George et al., 2017). Transcription factor NFE2L2 is an important regulator of autophagy gene expression. In AD patients, neurons expressing high levels of APP also expressed NFE2L2, indicating their attempt to degrade intracellular aggregates through autophagy (Pajares et al., 2016). Microglia activation and secretion of inflammatory cytokines are involved in the pathogenesis of AD, and STAT5B plays a crucial role in mediating IL-3-induced microglia activation (Natarajan et al., 2004). APP contributes to maintaining synaptic homeostasis (Müller et al., 2017), and wild-type huntingtin (HTT) facilitates the transport of APP by increasing the number of APP-containing vesicles (Colin et al., 2008). MAPK9 is an important player in the stress response and was reported to mediate autophagy induced by Aβ1-42 oligomers. In addition, studies have also elaborated on the important role of YWHAZ (Ho Kim et al., 2015; Yang et al., 2020; Zhou et al., 2020), PRKCD (Park et al., 2020), and SDHC (Bisht et al., 2020) in the development of AD. These findings in experimental studies strongly support our analytic results. Nevertheless, the molecular mechanism of these AGs contributing to AD pathogenesis is still poorly understood, and further studies are required to investigate the potential mechanisms of the AGs.

We observed that IGF1R, MXI1, PPARA, YWHAZ, and MAPK9 were remarkably strongly correlated with pathologic progression in AD and may function as facilitators or inhibitors of AD. There is no previous literature addressing the role of MXI1 in the development of AD. Mxi1 proteins has been proved to be essential in cellular growth control and in the induction of the differentiated state (Schreiber-Agus et al., 1998). Previous study identified Myc-Mxi1 signaling as a crucial downstream effector of FoxOs in the regulation of proliferation of renal cancer (Gan et al., 2010). Three key features of senescent cells include increased resistance to apoptosis death, a block-to-cell proliferation, and alterations in differentiated functions (Tsugu et al., 2000). It is likely that the studies probing the role of MXI1 in brain aging has significance for revealing potential pathogenesis of AD. These AGs have cell type-specific expression patterns and function. IGF1R is differentially expressed in oligodendrocytes and pericytes, MXI1 in pericytes, OPCs and microglia, PPARA in pericytes, YWHAZ in ECs and excitatory neurons, MAPK9 in ECs, excitatory neurons, and microglia. However, attentions was seldom paid to the function of these key AGs in a specific cell type. The problems become much more complicated owing to that these genes are shown to be dysregulated in different directions across different NVU cell types. Consideration for cell type-specific regulatory mechanism may be important in the design of therapeutic targets for AD. Further study is required to elucidate the cell-specific roles of these genes in the pathogenesis of AD. Our results provide valuable clues for investigating potential mechanism underlying AD.

## Conclusion

There is no effective therapy for AD at present; thus, further studies are needed to determine more accurate biomarkers with clinical utility. This study assessed aging-related transcriptomic changes in NVU cells of AD, which were validated with bulk transcriptome data. We identified 15 crucial DEAGs closely associated with AD by a series of bioinformatics analysis, including IGF1R, MXI1, RB1, PPARA, NFE2L2, STAT5B, FOS, PRKCD, YWHAZ, HTT, MAPK9, HSPA9, SDHC, PRKDC, and PDPK1. The diagnostic model based on the 15 AGs was constructed with relatively high AUC values in the external dataset. Of these, IGF1R, MXI1, PPARA, YWHAZ, and MAPK9 were remarkably strongly correlated with pathologic progression in AD and may be candidate genes for future molecular studies. More importantly, different expression patterns of key AGs in specific cell types may serve as a cell-specific therapeutic target for AD. Our comprehensive analysis of the snRNA sequence of NVU cells and bulk transcriptome data of AD reveals previously unknown molecular changes, as well as cellular targets, that potentially underlie the functional dysregulation of NVU in AD, providing important insights for therapeutic development.

## Data availability statement

Publicly available datasets were analyzed in this study. This data can be found here (http://www.ncbi.nlm.nih.gov/geo/), GSE174367, GSE48350, GSE122063, GSE29378, and GSE106241.

## Ethics statement

The data relating to participants were obtained from the public GEO database. Ethical review and approval were not required for this study on human participants in accordance with the local legislation and institutional requirements. Written informed consent was not required in accordance with the local legislation and institutional requirements.

## Author contributions

YZ wrote the manuscript and drew the figures. Y-ZX provided methodological advice. Y-SL conceived the idea and had been involved in manuscript conception and drafting. All authors read and approved the final manuscript.

## Footnotes

1
http://genomics.senescence.info/genes/
