# Supplementary figures and images for: Accelerated aging-related transcriptome alterations in neurovascular unit cells in the brain of Alzheimer’s disease
Source: Front Aging Neurosci. 2022 Aug 18;14:949074. doi: 10.3389/fnagi.2022.949074 (PMC9435434; doi:10.3389/fnagi.2022.949074)

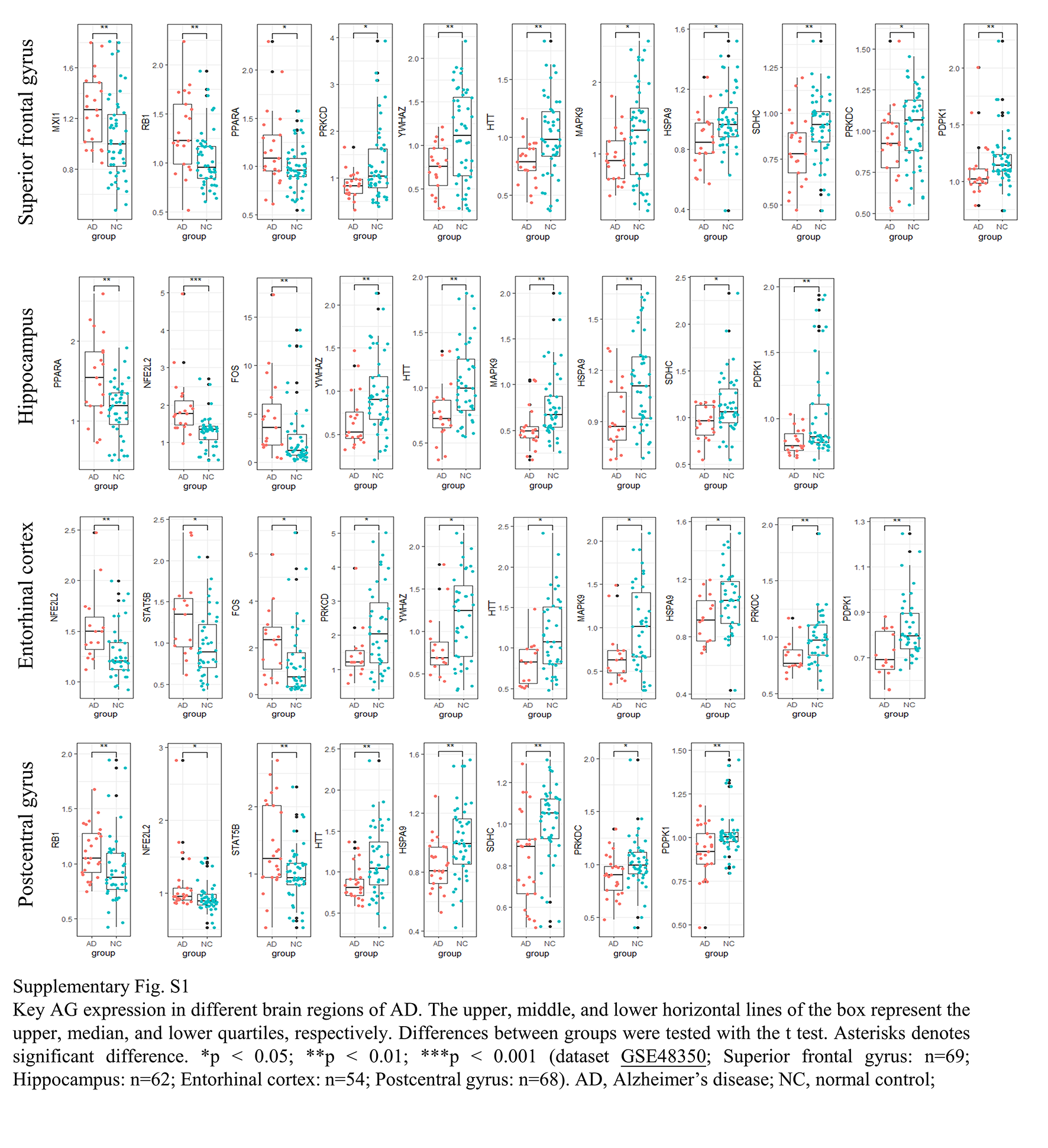

Supplement: Supplementary file 1 [file Image_1.TIF]
